# Asphalt Mixtures Fatigue Life Considering Various Environmental Impacts

**DOI:** 10.3390/ma16030966

**Published:** 2023-01-20

**Authors:** Eryk Mączka, Piotr Mackiewicz

**Affiliations:** Faculty of Civil Engineering, Wrocław University of Science and Technology, 50-370 Wrocław, Poland

**Keywords:** environmental factors, road salt, water and frost, degradation, mineral asphalt mix, asphalt concrete, fatigue life

## Abstract

The pavement structure during the colder seasons (winter) or in regions located above sea level is commonly affected and deteriorated by many environmental factors. Two prominent factors are water and frost (weather) or road salt (maintenance). According to the article’s literature review, there are only a few studies related to water and frost or road salt impact on mineral asphalt mixes considering fatigue. Most of the tests were performed on mixes containing common road asphalt or only one binder content level was investigated. There are no articles that investigate this problem comprehensively including new asphalt, its content levels, or production technology. Based on the literature review, the main problem regarding degradation impact on mixtures fatigue life was stated. The investigation was performed using two proprietary experimental methods allowing approximates in situ conditions regarding environmental impacts. A dynamic four-point bending fatigue test was applied to evaluate degradation considering fatigue. The investigation was performed using four coarse-graded asphalt mixtures (asphalt concrete AC 22) which differed in binder type (35/50 WMA, 35/50, 25/55-60, and 25/55-80 HIMA), content level (4.24%, 4.03%, 3.82%), and production technology (hot and warm). Regarding the results obtained, the authors proposed a degradation ratio regarding fatigue life variability. Based on the obtained results and ratio used, it was found that both interactions caused a significant fatigue life decrease—in the worst case, over tens of percent. Furthermore, it was found that asphalt mixture resistance to environmental factors depends on binder type, its content level, air void content, and discussed impact. Moreover, asphalt mixtures’ susceptibility to degradation (fatigue) is extreme at lower binder content levels and accelerates due to air void content increase. In the article, it was also stated that the highest resistance was reached by a mixture with highly modified asphalt (25/55-80 HIMA). It was also found that the SBS polymer dosage increase in the asphalt matrix enhances asphalt mixture resistance to environmental impacts. The least resistant to the environmental degradation mixture was WMA (35/50 WMA).

## 1. Introduction

Water and frost are common environmental factors affecting road structure made from, e.g., mineral asphalt mixes (MMA)—especially during the winter season. Various chemicals and mixtures (including treatment systems) are used to prevent or minimize their impact on vehicle movement (traffic conditions). One is road salt—sodium chloride (NaCl), an effective agent used in winter maintenance [1,2,3,4,5,6,7,8,9,10]. Although road salt application is an effective treatment for ice removal, maintenance procedures require cyclical repetition. Unfortunately, it is the main reason for chemical accumulation in the road lane and surrounding terrain which causes serious issues to the pavement and its condition.

Pavement structure deterioration is a complex problem. Mechanical forces (vehicle wheels) and environmental impacts such as water, frost, and road salt degrade the utilized material. All of them negatively affect pavement strength and durability. The final impact consequences are issues such as cracking, chipping, and others. Furthermore, environmental impacts are seasonal. They could accumulate through all years of the construction’s use, reducing their durability.

Water, frost, and road salt might impact pavement in various forms, e.g., rainwater, a mixture of brine solution + rainwater through freeze-thawing, or chemical reactions (mainly road salt interacting with the material surface) connected with enthalpy changes. Furthermore, rainwater and brine solution might affect the surface course as well as deeper layers. Liquid or solution might penetrate the construction in many different ways, detailed in [11,12,13].

Although all factors deteriorate pavement construction, only the mechanical one is currently considered in laboratory test procedures or pavement design guidelines. This is a severe problem leading to underestimating asphalt mixtures’ durability (fatigue life) utilizing in pavement construction. Due to the aforementioned problem, it could be emphasized that the influence of environmental factors on asphalt mixture’s strength and durability is a new topic and deserve a deeper investigation.

Environmental impact on asphalt mixtures is not an entirely new subject area. Many publications such as [14,15,16,17,18] present the negative influence of the water-frost factor. The cited authors tried to investigate the degradation mechanism due to water-frost impact. It was emphasized that damage caused by this factor is related to air void content, its location in the structure, and retention (liquid ability to penetrate) via asphalt mixture.

On the other hand, road salt impact is hardly ever discussed. Only a few key publications [12,13,17,18,19,20] are related to asphalt mixture degradation considering the salt impact. Cited articles primarily focused on degradation evaluation via indirect static tensile (ITT) based on material strength. Three or four-point bending dynamic tests for stiffness modulus variability estimation were rarely used.

Although the environmental impacts are essential, asphalt mixture degradation considering these adverse effects in meaning, e.g., fatigue life, has not been effectively examined so far.

A complete and current literature review regarding the asphalt mixture degradation problem considering water-frost and road salt impact was presented in the previous manuscript [11]. The paper extensively discussed performed attempts, tests, methods used, and current trends in MMA testing. It was emphasized that many authors used mainly static indirect tensile tests (ITT)—there were no fatigue tests applied (except low cycle indirect tensile fatigue test ITFT). In addition, a few studies were listed using tests other than the overmentioned one to assess degradation, such as 4-PB-PR (which corresponds with pavement layer behavior under load).

In the performed review, various combinations of freeze-thaw cycles (water and frost) or soaking in a salt solution for several days (mainly NaCl concentrations of 5, 10, and 15%) performed by cited authors were also discussed. It was emphasized that the studies had not analyzed the conditions of de-icing agents’ impacts for longer (than 7 days) or higher levels of brine concentration (especially those that may occur in winter after intensive pavement salting, ex., 20% or 25%). Moreover, the subject area was not investigated as a complex problem—water-frost or road salt degradation regarding the same asphalt mixes and testing conditions (allowing for the comparison between negative impacts).

The review also states that attention is currently paid primarily to mixtures intended for the surface course or binding course of the AC 13 or AC 16 type, containing common 50/70 or occasionally modified 45/80-55 asphalt. No studies have concerned coarse-grained mixtures, such as AC-22, intended for the base course or binding course (where the degradation caused by water, frost, or salt may also occur). In addition, no articles comprehensively address the topic of asphalt mix degradation caused by environmental factors based on different types of binder (common, modified, high-modified) and production technologies (e.g., hot technology—HMA and warm WMA). Furthermore, there is a lack of articles that analyze environmental impacts on mixture durability (fatigue life).

In the previous manuscript [11], degradation based on stiffness modulus changes was verified and evaluated by applying the proposed degradation ratio $DI_{S}$. It was found that the degradation of asphalt mixtures caused by environmental effects is significant (stiffness modulus decreased by over a dozen percent). It also depends on binder type and additives allowing temperature production technology reduction. Mixes containing modified or highly modified asphalt were much better resistant to stiffness modulus changes than common ones. Observed modulus changes of all mixes were used to verify how pavement durability would change (regarding stiffness modulus mix changes via water-frost or road salt impact). To obtain the aim, the AASHTO fatigue criterion was applied. It was noticed that pavement durability could decrease by a few dozen percent.

**The current manuscript is an extension (sequel)** of the asphalt mixtures degradation subject area discussed in the previous article [11]. The main focus of the following paper is placed on asphalt mixtures’ fatigue life, considering degradation caused by environmental impacts (water-frost or road salt) intended for binder course and base course (e.g., AC 22 W or AC 22 P).

Following the aforementioned observations, the main aim of this study was stated. **The manuscript’s main goal is** to evaluate the negative environmental impact (water and frost or road salt) on the asphalt mixture’s fatigue life based on the experimental method. The manuscript concern coarse-graded asphalt concrete (AC 22) mixes that might be applied to the binder course or base course of pavement construction layers.

In the article, four asphalt mixes are investigated and compared. They varied in:

- binder type (four binder types),
- content level (three levels, the same content for each set of four mixes),
- production technology.

## 2. Materials and Methods

Four road mixes, course-graded asphalt concrete (AC 22), intended for the binding and base course layers of flexible construction, were designed. Four various asphalt types were applied into mixes. Three various binder content levels were also investigated. The asphalt binder was selected based on a similar penetration index and usage frequency in road construction. The mineral mix curve was identical per mix. The stone material from the same source was used.

Such assumptions mentioned above allowed us to analyze selected asphalt mixtures effectively and in the complex.

Compared mixtures varied:

- the type of binder,
- content level,
- production technology (hot HMA and warm WMA production technology).

Table 1 presents the asphalt mixture’s composition intender for experimental fatigue tests. The designed mix recipes’ usefulness was checked following the requirements included in the regulation on Polish roads, considering the EN standards [21,22,23,24,25]. All mixtures met the basic requirements.

Prepared recipes and materials were named according to the following convention:

**Referred to mixture and recipe**:

- A i—mix containing 35/50 WMA binder (warm technology), i—number of recipe (binder level content),
- B i—mix containing 35/50 binder (hot technology), i—number of recipe (binder level content),
- C i—mix containing 25/55-60 binder (hot technology), i—number of recipe (binder level content),
- D i—mix containing 25/55-80 HIMA binder (hot technology), i—number of recipe (binder level content).

**Referred to mixture recipe and binder level content**:

- mix A–D, recipe number 2 – 4.24 [%]—input binder content,
- mix A–D, recipe number 3 – 4.03 [%]—the first level of binder content optimization,
- mix A–D, recipe number 4 – 3.82 [%]—the second level of binder content optimization.

As this manuscript is a **sequel** of [11], **significant elements and assumptions** from the proprietary experimental method **are reminded below**.

Prismatic beams (65 × 65 × 390 mm) were prepared for the fatigue tests. Each samples side was polished. The prepared mixtures were appropriately selected to create 3 sample sets per mixture and asphalt level (6 beams per set with similar stiffness modulus—estimated using the 4-PB-PR stiffness modulus test [26]). The following sets were named:

- reference (ref),
- subjected to water and frost impact (f-t),
- subjected to water and frost impact road salt (rs).

**Figure 1:**
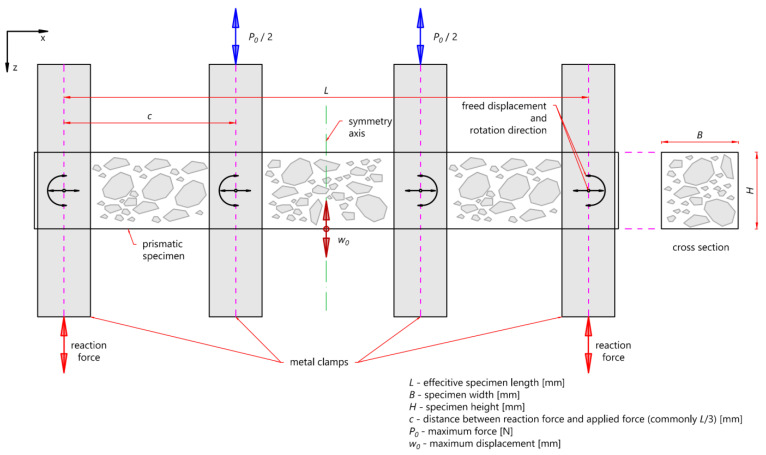
Static diagram of the 4-PB-PR test.

The PN-EN fatigue standard [27] was applied to perform laboratory tests. The dynamic four-point bending (4-PB-PR) fatigue test was used to achieve the paper’s main aim. The test method diagram is presented in Figure 1.

Prismatic beams analyzed in the article [11] were re-used in the fatigue test part. The general test parameters are emphasized in Table 2.

**Figure 2:**
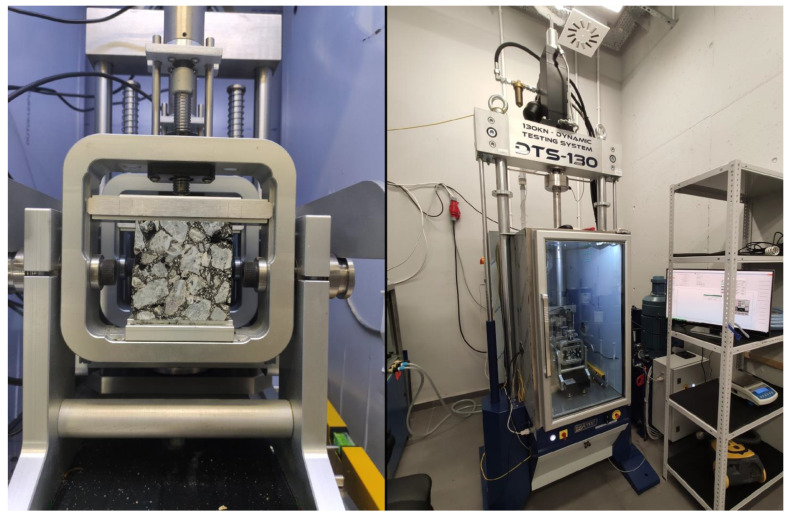
Hydraulic tester machine view.

The fatigue tests were made in a hydraulic high-load tester (*Dynamic Testing System* 130 kN) dedicated to asphalt mixtures. The device was equipped with a climatic chamber, a mounting frame (4-PB), a digital controller, and appropriate sensors, e.g., displacement, temperature, and force. The machine configuration (e.g., PID parameters) was equal to the test performed in the article [11]. The machine and a sample on the test stand are presented in Figure 2.

Variables simulating in situ conditions were applied in the study to investigate the asphalt mixture degradation caused by environmental impacts. The research was carried out using two proprietary experimental methods extensively characterized in [11,13]. The main assumptions cross-referred to cited articles are presented below.

### 2.1. Water and Frost Impact (Freeze-Thaw Cycle)

The first proprietary method is based on the Indirect Tensile Strength Ratio (ITSR) described in the Polish standard WT-2 [22] and the American standard AASHTO T-283 2021 [34]—a commonly used static test according to water-frost impact.

Instead of using ITT, the 4-PB-PR test was applied. Some general test assumptions were modified. The following modifications are presented below.

- The beams were thoroughly soaked in water; the vacuum suction procedure was skipped.
- The container with water and specimens was located directly in the thermal chamber. Full programmed temperature ramp and automation of the whole conditioning process were guaranteed. Initially, for 72.0 h, the samples were conditioned at 40.0 °C; then, for 24.0 h, they were frozen at (−) 18.0 °C. Temperature-reaching time was also considered.
- The environmental impact was assessed based on the fatigue life variability—freeze-thaw (f-t) set of samples regarding the reference (ref) set.
- The target test temperature was 10.0 °C instead of 25.0 °C (fatigue life evaluation according to EN and WT-2 standards).
- The method D (geometric measurements) was applied to determine the bulk density [35]; it was recognized as effective [21] for polished specimens,
- After defrosting and reaching the test temperature, the tested samples:
  - were removed from the container,
  - successively dried using a microfiber cloth,
  - the test start was carried out instantaneously (the preparation process did not exceed a minute).

**Figure 3:**
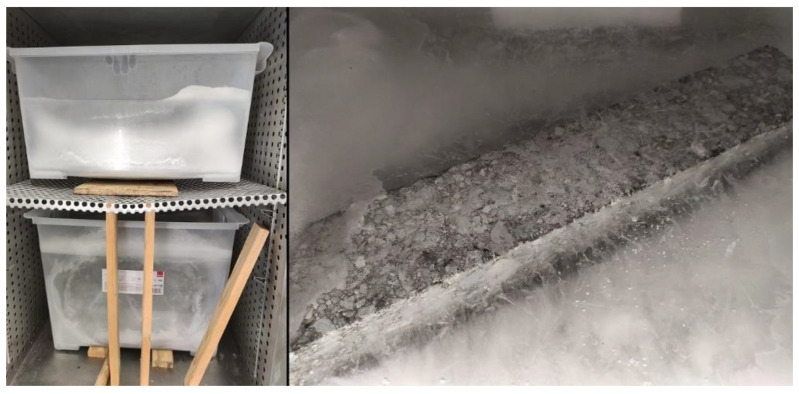
Camber view: water-frost impact [11].

The chamber view was presented in Figure 3.

### 2.2. Road Salt Impact: Brine Soaking

The second experimental method was related to the study of the impact of road salt on asphalt mixes. There are no developed test procedures for evaluating road salt impact on MMA currently.

The selected specimens were placed in a closed container. Inside the container, four independent water pumps were installed. An average pumping capacity was 7500 l/h per pump. Pumps forced solution circulation. The application of these devices was aimed at two crucial aspects:

- the brine concentration was constant (no local concentration points),
- forced circulation causes cyclical solution pressure on the material (approximation of in situ conditions—the vehicle wheel forces the brine mixture into the pavement (e.g., via cracks, chippings) under pressure).

**Figure 4:**
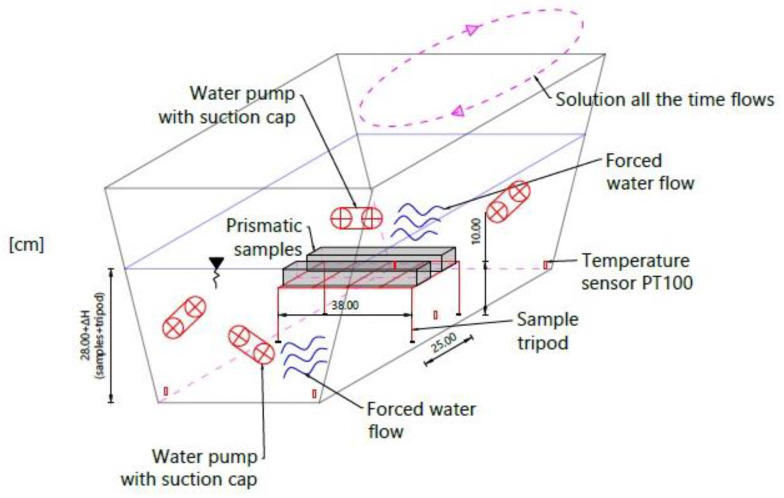
Experimental brine soaking pattern [11].

**Figure 5:**
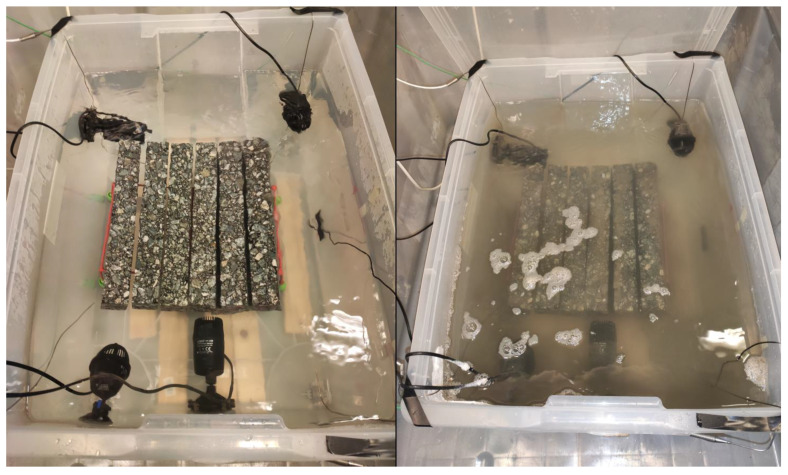
Armed container before (**left hand side**) the road salt dosage and after experiment (**right hand side**).

Furthermore, the temperature sensors were installed in five places near the bottom of the container. It made it possible to track temperature changes. The experiment test stand and its mechanism are presented in Figure 4 and Figure 5.

The brine solution concentration was equal to 20.0%. The 20% brine concentration was chosen as a compromise between saturated and unsaturated solutions, which could affect pavement construction.

The final conditioning temperature was 10.0 ± 0.2 degrees Celsius. It is one of the equivalent temperatures in Poland that can be used in the design of flexible pavement structures [29,30]. Moreover, this temperature level is commonly used in stiffness and fatigue life tests, following European and Polish national guidelines or PN-EN standards [22,24,25,36]. Its appliance allowed to standardize testing procedure and compare impacts due to fatigue.

**Figure 6:**
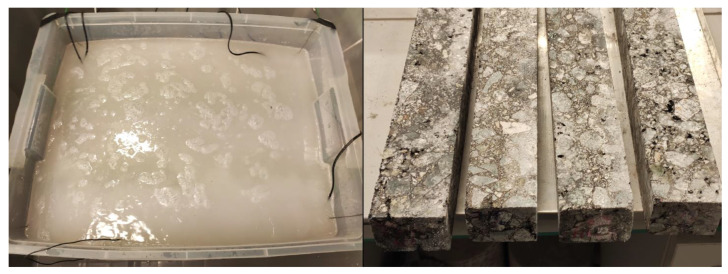
Brine system and specimens after soaking.

The beams were soaked for 2 weeks. The rules adopted in the experimental research method were established based on winter condition observations, and the primary maintenance practices [7,8,9] applied in European countries such as Poland. Brine system and specimens after soaking are presented in Figure 6.

It should be emphasized that the water and frost or road salt impact might be lower or even comparable for deeper layers of pavement construction in comparison to the surface course. It strictly depends on, e.g., the current pavement condition (damage status), region for the desired road (climate), or other variables and construction site processes (vehicles sometimes drive through unaccomplished pavement—the binder course or base course directly cause fatal damage to these layers [37], especially during winter season). In such cases, the pavement deterioration process is a unique and complex problem.

## 3. Results

### Laboratory Test Results

**Figure 7:**
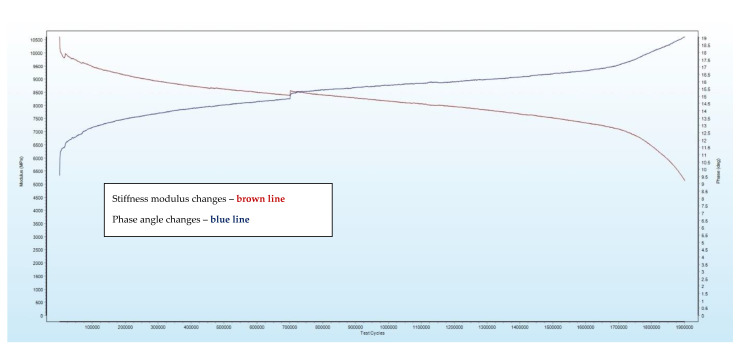
Exemplary sample result registered by testlab software.

Considering all the assumptions mentioned above, the fatigue life was estimated based on the PN-EN 12697-24:2018-08 standard [27]. Arbitrary fatigue criterion (initial stiffness modulus decrease by 50%) was applied. The results were recorded in real-time using the Testlab software. An exemplary result is presented in Figure 7.

Based on references [13,22,28,30,38], two test strain levels considering analyzed materials were estimated. The initial binder content level (4.24%) was chosen as a reference to determine the final strain level to fatigue tests for all mixes A–D. The following strain amplitude levels were analyzed:

- **130 με (initially).** The typical value could be archived in pavement construction layers (especially in colder seasons). It was applied to check all asphalt mixtures’ vulnerability to mechanical factors. Mixes with polymer binders are resistant, especially for higher binder content levels. They will not achieve the fatigue criterion point at too low strain values due to horizontal leveling of stiffness modulus changes (such mixes start to be unsusceptible during the test). Finally, they require a very high (infinite) amount of test cycles to obtain the criterion, if it is even possible). In this case, **mixes A and B** have **reached the fatigue criterion**, and in mixes, **C and D (with SBS polymer), not**—the stiffness modulus leveled horizontally at 85% and stopped decreasing. **Results for this strain level were rejected** and will not be discussed.
- **200 με (finally).** Value was applied **to comprehensive tests** due to reaching for all mixes chosen fatigue criteria. All mixes (A–D) have reached the fatigue criterion. Furthermore, the value applied allowed accelerating tests (one specimen test took several days). The strain value could be achieved in pavement construction layers (especially in warmer seasons or when the traffic impact is high (overloaded vehicles)).

**The comprehensive fatigue** tests referring to degradation caused by environmental impacts considered all chosen binder content levels and impacts (ref, f-t, rs) for designed mixes (A–D).

Based on the test results, a degradation ratio referring to fatigue life changes was developed. The formula was presented in Equation (1). It expresses a change in the fatigue life caused by an appropriate impact, such as water, frost, or road salt. The degradation ratio made it easier to analyze asphalt mixture degradation considering fatigue.

(1) $$DI_{\left(F\right)}=\left(\frac{E_{50\%,2\;}^{*}}{E_{50\%,1\;}^{*}}-1\right)\cdot 100\%$$

where:

$DI_{\left(F\right)}$—degradation ratio expressed by asphalt mixture’s fatigue life change [%],

$E_{50\%,\;1\;}^{*}$—average fatigue life (in cycles, for the samples set) regarding the reference set [MPa],

$E_{50\%,2\;}^{*}$—average fatigue life (in cycles, for the samples set) regarding the corresponding impact (degraded) [MPa].

Test results due to the excessive data are presented and discussed as an average of six markings (independently tested beams) per each mix recipe. One additional sample test was performed on each specimen set (in reference to [11]) due to fatigue life variability. The dispersion of the results (expressed by the classical coefficient of variation), considering the specificity of the method used (the four-point bending test (4-PB)), was low—it did not exceed 8.5%. The data are presented in Table 3.

The following symbols are used in Table 3:

ref—reference set,
f-t—set impacted by water and frost,
rs—set impacted by road salt,
$V_{b}$—the percentage of binder content added to MMA (by weight) [%],
$V_{m}$—the air void content in MMA [%],
$E_{0,F}^{*}$—average initial stiffness modulus per mix set [MPa],
$E_{50\%,F}^{*}$—average final stiffness modulus per mix set (after reaching fatigue criterion) [MPa],
$N_{F,E50\%}$—average fatigue life per mix set referring to arbitrary criterion [cycles],
$DI_{\left(F\right)}$—degradation ratio expressed by asphalt mixture’s fatigue life change [%].

Four-point bending (4-PB-PR) fatigue test results () expose the asphalt mixture degradation caused by freeze-thaw and road salt impact. At first glance, the environmental impact caused significant changes in initial stiffness and primarily fatigue life. The degradation level was observed to increase with air void content in the mix and with a decrease in binder content level. It is worth mentioning that selected and compared mixed differs only in binder type and production technology. Results are comprehensively analyzed in the discussion part.

## 4. Discussion

The article focused on asphalt mixture degradation caused by environmental impacts, e.g., water, frost, or road salt. Although the problem primarily referred to fatigue life, other crucial asphalt mixture parameters and correlations were also discussed. It was stated that environmental effects impact mixtures significantly. The dynamic 4-PB-PR fatigue test and arbitrary fatigue criterion were applied to investigate mixes resistance to such effects. Two proprietary experimental research methods using mentioned dynamic test were reminded from previous publications [11,12,13]. The methods used allowed one to effectively determine the material degradation caused by environmental impacts through the fatigue life variability. Four types of mixtures, differing only in the binder type (common asphalt 35/50 WMA and 35/50, modified 25/55-60 (SBS polymer), and highly modified 25/55-80 HIMA (SBS polymer)) and technology production (WMA—warm, HMA—hot), were prepared for the research. Three identical binder content levels were also analyzed—starting level (4.24%) and two optimization levels (4.03%, 3.82%). A degradation ratio referring to fatigue life was developed based on the obtained results.

Furthermore, the degradation ratio referring to stiffness modulus [11] variability was also used and fit according to the fatigue test specificity. Its value made it easier to assess and analyze the degradation caused by environmental factors through all mixtures. All assumptions and tests allowed compare mixes effectively and complexity.

According to the results obtained in the previous section (Table 3), some crucial observations were found. The following observations related to asphalt mixture degradation considering fatigue are listed below.

**Figure 8:**
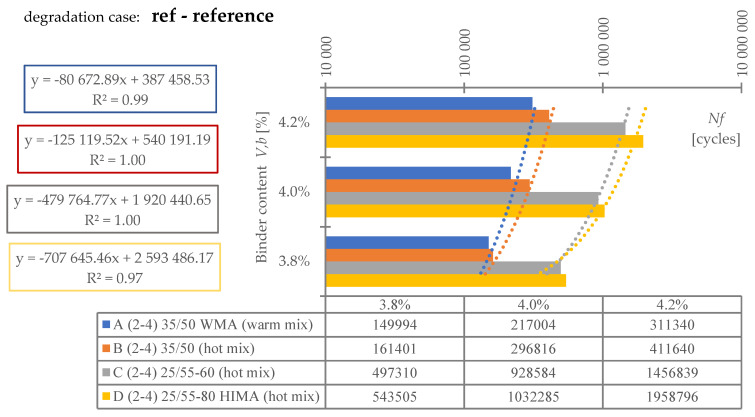
Fatigue test results—reference set.

**Figure 9:**
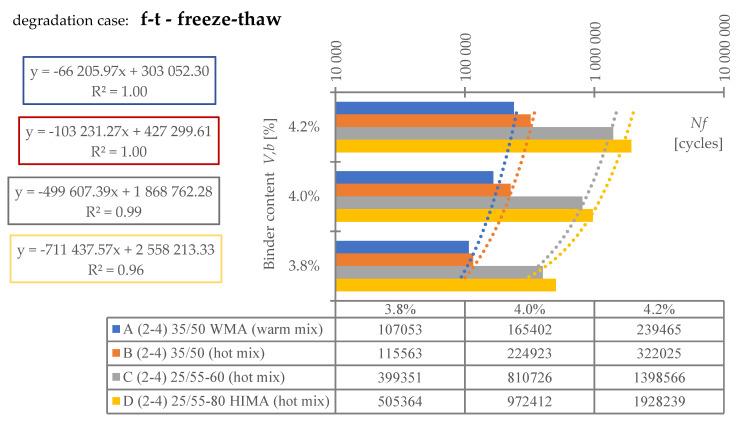
Fatigue test results—water-frost impact.

**Figure 10:**
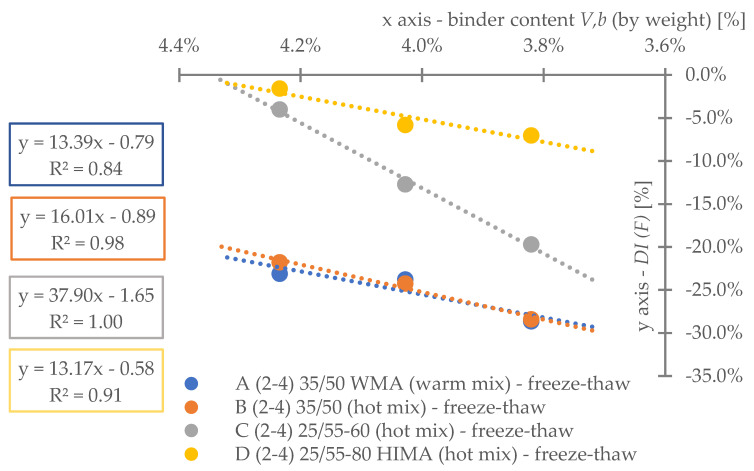
Degradation ratio variability (fatigue) through all mixes (water-frost).

**Figure 11:**
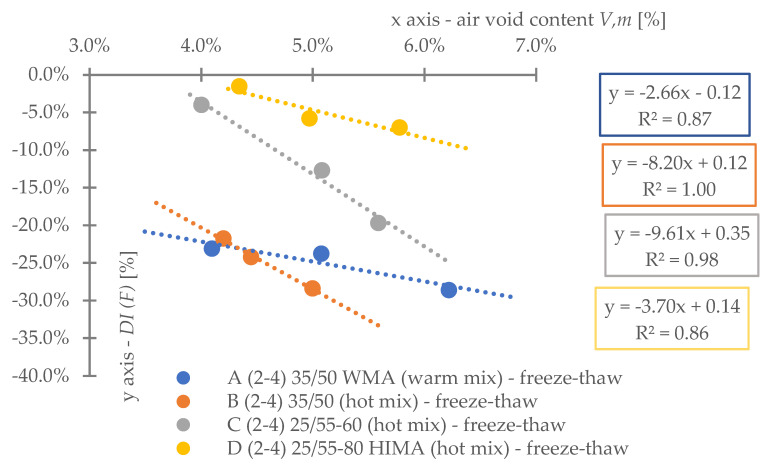
Water and frost fatigue degradation considering air void content variability.

**Figure 12:**
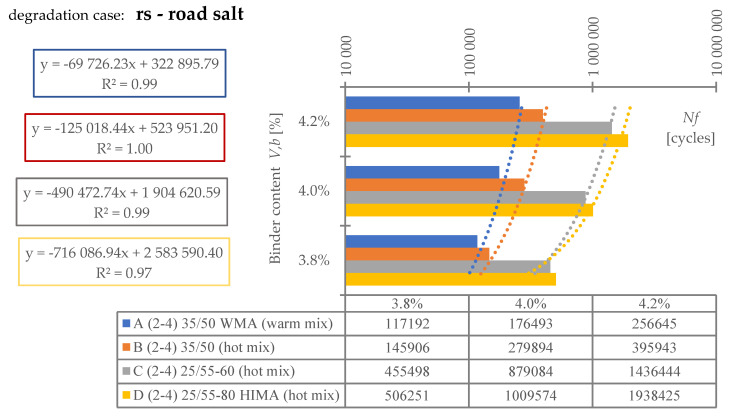
Fatigue test results—road salt impact.

**Figure 13:**
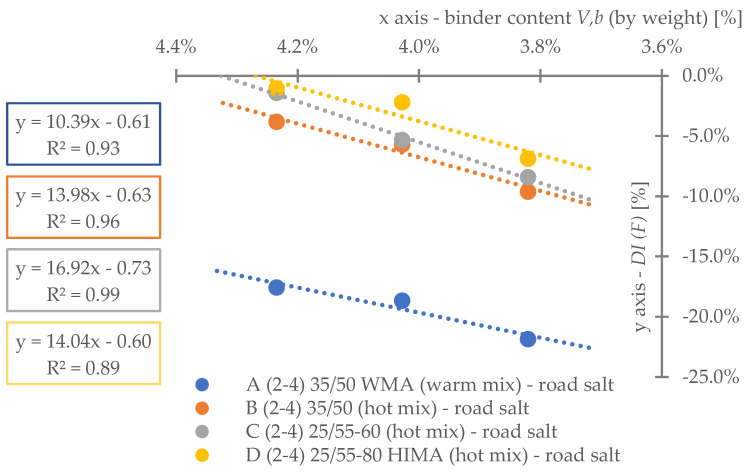
Degradation ratio variability (fatigue) through all mixes (road salt).

**Figure 14:**
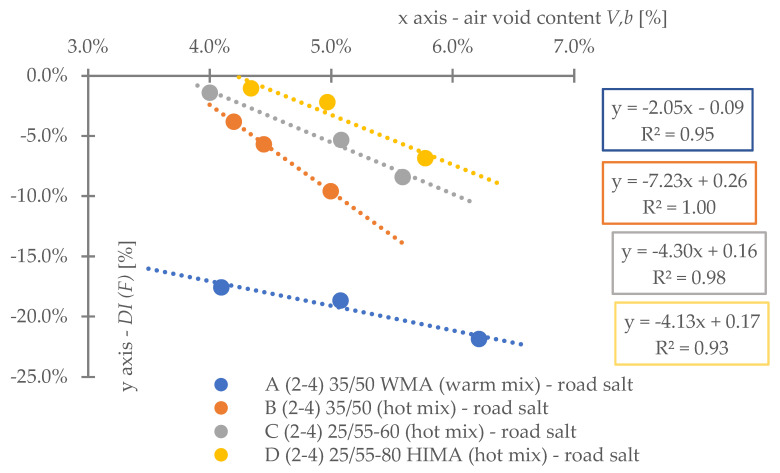
Road salt fatigue degradation considering air void content variability.

1. Regarding **reference set test results** (Figure 8), the highest durability, regardless of binder content level, was characterized by mix D (25/55-80 HIMA, HMA). Next was mix C (25/55-60, HMA, lower SBS polymer content in the matrix than D). Sequentially, B (common 35/50, HMA) and A (common 35/50 WMA, WMA). Differences between mixes’ durability were significant—mixture D was even six times more durable than A (at 4.2%). Differences between mixes fading with a decrease of analyzed binder content level—mix D vs. A is only 3.6 times more durable at 3.8% (decrease approximately by half).
2. Based on (Figure 8) it is stated, asphalt matrix modification by polymer SBS increases mixture fatigue life significantly. Moreover, as the modification is higher (higher SBS content), better mixture fatigue resistance is archived. Comparing mix D vs. C, fatigue life extension raised even 35% (at $V_{b}$—4.2%) but downgraded to 9% (at $V_{b}$—3.8%).
3. Binder content impacts mixture fatigue life more than asphalt matrix modification (adding SBS additive).
4. Observing (Figure 8), all mixtures (A–D) fatigue life was decreased with binder content reduction (regardless of the analyzed level). Changes were significant—a fatigue life decrease between content levels in the worst case was even 53%. Furthermore, the most susceptible mix to such changes was D (with highly modified asphalt). It is stated that an increase in SBS polymer in matrix could cause asphalt mixture to be more dependent on binder content level.
5. A linear regression model (Figure 8) could be used to predict fatigue life for discussed mixes and binder content levels successfully. The model determination coefficient was very high—0.97 ($R^{2}>0.97$). The result could also prove that all technology assumptions and efforts for mixes were appropriate—homogenous and comparable sample sets due to fatigue were produced at each binder content level.
6. Comparing mixes C and D regression model angle (Figure 8), it is stated that the effect of asphalt matrix modification is the most visible at higher binder content value. The relative speed of changes due to fatigue life increase was about 47% higher for mix D in comparison to C.
7. Asphalt mixtures containing common road asphalt (35/50) (Figure 8) were comparable due to fatigue life at each binder content level. Although results were comparable, mix A, made in warm (WMA) production technology, was approximately 35% worse due to fatigue life speed changes considering binder content level (linear regression model angle ratio) in comparison to B, made in hot technology (HMA). Asphalt mixtures made in hot technology might provide better (up to 32% for at $V_{b}$—4.2%, B vs. A) resistance to fatigue than warm ones.
8. Regarding test results referring to the impact of **water and frost** (environmental) (Figure 9) and comparing it with (Figure 8), it is stated that such an effect caused fatigue life reduction among all mixes.
9. A linear regression model (Figure 9) could be used to successfully predict fatigue life for discussed mixes and binder content levels—including water-frost impact. The model determination coefficient was very high—0.96 ($R^{2}>0.96$). Comparing the model’s angles (Figure 8 and Figure 9), its value was changed. It might be stated that water and frost negatively impact asphalt mixtures’ speed changes through different binder content levels (degradation was accelerated). It is mainly dependent on a binder type.
10. According to degradation (fatigue life decrease—$DI_{\left(F\right)}$ ratio) caused by water-frost impact (environmental) (Figure 10), it is significant. In the worst case, its value was equal to 28.6% (mix A4, $V_{b}$ = 3.82%).
11. Observing (Figure 10), degradation caused by water and frost impact depends on the asphalt mixture binder type and its content level. $DI_{\left(F\right)}$ ratio differs from 1.56% (D2, $V_{b}$ = 4.24%) to 28.63% (A4, $V_{b}$ = 3.82%).
12. Among all the tested materials (Figure 10), the lowest sensitivity to water and frost was represented by the mixture D with HIMA (highly modified asphalt 25/55-80)—a decrease in durability by about 1.56% at $V_{b}$ = 4.24% and 7.02% at $V_{b}$ = 3.82%. Next is C (modified asphalt 25/55-60), where its durability reduction was about 4.00% at $V_{b}$ = 4.24% and as much as 19.70% at $V_{b}$ = 3.82%. In sucession was the mixture B (35/50)—durability decrease was about 21.77% at $V_{b}$ = 4.24% and 28.40% at $V_{b}$ = 3.82%. Ultimately, the last was mixture A (35/50 WMA). The durability decreased by about 23.09% at $V_{b}$ = 4.24% and 28.63% at $V_{b}$ = 3.82%. It is stated that asphalt matrix modification (by applying SBS polymer) allows increasing mixture resistance to degradation caused by water and frost on the mixtures effectively.
13. A comparative analysis between mixes C and D (Figure 10) showed that polymer dosage increase (in asphalt matrix) enhanced mix resistance to water and frost from 2.56 to 2.81 times ($DI_{\left(F\right)}$ ratio comparison through binder content levels). As more SBS polymer content is in the mix, even better water-frost resistance might be achieved.
14. Comparing mixes inside hot technology (HMA—B, C and D) via $DI_{\left(F\right)}$ ratio, mix D (highly modified bitumen) characterized 14 times ($V_{b}$ = 4.24%) and about four times ($V_{b}$ = 3.82%.) and C (modified bitumen)—about five times ($V_{b}$ = 4.24%) and about 1.44 times ($V_{b}$ = 3.82%.) lower decrease in fatigue life caused by water and frost impact than mix B (common bitumen). Compounds C and D are more durable than B, making them universal for use on virtually any road in terms of intensity.
15. Comparing mixes through different technologies (WMA represented by mix A vs. HMA, B)—the mixtures in the HMA (B) technology were characterized as more resistant to water and frost than WMA (A) by approximately 9%. It is stated that mixes produced in the WMA technology might be an alternative to HMA on roads with low traffic and more complex environmental conditions (water-frost). Although degradation caused a similar effect, its value is relatively high. $DI_{\left(F\right)}$ ratio varies between 21.77% (B2) and 28.63% (A4). It is about 25% of the reference fatigue life. The impact of water and frost to asphalt mixtures requires deeper investigation considering fatigue, especially for mixes containing common road asphalt.
16. A linear regression model (Figure 10) could be used to successfully predict the degradation effect (to water and frost) resulting in fatigue life decrease. The model determination coefficient was high (A, D, $R^{2}>0.84$) and very high (B, C—$R^{2}>0.98$).
17. According to applied linear regression models (Figure 10), degradation speed (fatigue) characterized by a model angle is similar in mixes A, B and D. Mix C is more vulnerable (averagely 2.64 times) to water and frost degradation through different binder content levels. It might be connected with air void content levels.
18. Moreover, comparing regression models’ angles (Figure 10), it was observed that models are shifted to each other. The angle is similar but the $DI_{\left(F\right)}$ ratio is not. Shift characterizes mix resistance due to water and frost (fatigue) through different binder types and a content level applied into the mix (as a degradation speed). Degradation speed and vulnerability are technically the same for mix A and B (models are superimposed)—influenced mainly by water frost impact. Mix C behaves slightly differently—degradation speed is higher but still is better than A and B due to $DI_{\left(F\right)}$ ratio. Mix D is characterized by a low angle value and $DI_{\left(F\right)}$ ratio—making it the best of all mixes due to this environmental impact.
19. A linear regression model (Figure 11) referred to air void content level and $DI_{\left(F\right)}$ ratio are well correlated. The model determination coefficient was high (A, D—$R^{2}>$ 0.86) and very high (B, C—$R^{2}>$0.98). It could be stated that degradation (fatigue) caused by water and frost also depends on air void content in the mix (except binder type and content). It is a similar observation to the articles [14,15,39] which focused on asphalt mixture strength degradation.
20. Analyzing the correlation between air void content level and $DI_{\left(F\right)}$ ratio (Figure 11) proves that the observation mentioned in point number 17 is accurate. The C mix regression model angle is the highest. Although mix B angle is at a similar (15% lower) angle value than C, if we consider air void content level for all binder contents, we might observe that mix B tended to be compacted more easily than C.
21. Regarding test results referring to **road salt impact** (environmental) (Figure 12), it also caused fatigue life reduction among all mixes. Moreover, comparing results with water-frost impact (environmental) (Figure 9) and reference (Figure 8), it is mentioned that road salt caused a slightly lower fatigue life decrease (in comparison to water-frost impact). Moreover, degradation caused by salt against water and frost ($DI_{\left(F\right)}$ ratio, Figure 13) in extreme cases: A (WMA)—is about 25% weaker (A4), B—is about 33% weaker (B4), C—is about 42% weaker (C4), and D—is about 3% weaker (D4) (comparable). The analyzed common road asphalts used in the mixtures characterized greater sensitivity to the negative impact of water and frost than road salt, similarly modified bitumens, while highly modified bitumens—similarly.
22. A linear regression model (Figure 12) could be used to successfully predict fatigue life for discussed mixes and binder content levels—including road salt impact. The model determination coefficient was very high—0.97 ($R^{2}>0.97$) among all mixes. Comparing the model’s angles (Figure 8 and Figure 12), its value was slightly changed. It might be emphasized that road salt negatively impacts asphalt mixtures’ fatigue life speed changes through different binder content levels. The highest change value referred to mixture A (common road asphalt 35/50 WMA), and it was equal to (-)14% (fatigue life speed slowed). Other mix’s model angles did not vary from reference significantly. Road salt slows down (limits) mixture resistance to fatigue crucially, especially for high binder content levels. Furthermore, it might be highlighted that due to the following observations, degradation via road salt impact depends on a binder type.
23. According to degradation (fatigue life decrease—$DI_{\left(F\right)}$ ratio) caused by road salt impact (environmental) (Figure 13), it is significant. In the worst case, its value was equal to 21.9% (mix A4, $V_{b}$ = 3.82%).
24. Observing (Figure 13), degradation caused by road salt impact depends on the asphalt mixture binder type but also its content level. $DI_{\left(F\right)}$ ratio differs from 1.04% (D2, $V_{b}$ = 4.24%) to 21.9% (A4, $V_{b}$ = 3.82%).
25. Among all the tested materials (Figure 13), the highest resistance to road salt impact was represented by the mixture D with HIMA (highly modified asphalt 25/55-80)—a decrease in durability by about 6.85% at $V_{b}$ = 3.82%. Next is C (modified asphalt 25/55-60). Durability reduction was about 8.41% at $V_{b}$ = 3.82% (mix resistance was about 22% lower than mix D at $V_{b}$ = 3.82%). In sucession was the mixture B (35/50)—durability decrease was about 9.60% at $V_{b}$ = 3.82% (mix resistance was about 40% lower than mix D at $V_{b}$ = 3.82%). Ultimately, the last was mixture A (35/50 WMA). The durability decreased by about 21.9% at $V_{b}$ = 3.82% (mix resistance was about 319% lower than mix D at $V_{b}$ = 3.82%). It is stated that asphalt matrix modification (by applying SBS polymer) also allows increasing mixture resistance to degradation caused by water and frost on the mixtures effectively.
26. Comparing mixes D and C referring to polymer SBS content increase (fatigue life decrease—$DI_{\left(F\right)}$ ratio, (Figure 13)), it might also be mentioned that higher asphalt matrix modification allows producing more resistant mixes to road salt effect. Modification causes the same effect as mentioned due to water frost impact—resistance was grown. According to road salt impact, it was, respectively: from 22.4% at $V_{b}$ = 3.82%, up to 34.1% at $V_{b}$ = 4.24%. Moreover, the resistance enhancement is more visible at higher binder content levels.
27. Based on observation number 25, the rapid decrease in the durability of mixture A in relation to B results from the additives appliance and content level used in the mix, allowing for the production of MMA in warm technology (WMA). It is assumed that the binder enriched with such substances reacts more easily with road salt, leading to excessive mixture degradation.
28. Comparing mixes inside hot technology (HMA—B, C, and D) via $DI_{\left(F\right)}$ ratio (Figure 13), mix D (highly modified bitumen) characterized four times ($V_{b}$ = 4.24%) and about 1.5 times ($V_{b}$ = 3.82%) better resistance to road salt impact than mix B (common bitumen). Mix C similarly, but at ($V_{b}$ = 4.24%) C vs. B test results were comparable. Compounds C and D are more durable and resistant to deterioration issues than B, making them universal for use on virtually any road in terms of intensity, especially at lower binder content levels.
29. Comparing mixes through different technologies (WMA represented by mix A vs. HMA, B)—the mixtures in the HMA (B) technology were characterized as more resistant to road salt impact than WMA (A). The distinction between them via $DI_{\left(F\right)}$ ratio (Figure 13) resulted, respectively: 4.6 times ($V_{b}$ = 4.24%), 2.27 times ($V_{b}$ = 3.82%). Furthermore, it is worth highlighting that considered WMA mixes fatigue life decrease is almost 24% of the reference durability of this MMA. It is stated that WMA mixes containing common road asphalts tend to be very vulnerable to road salt degradation. The impact of water and frost on asphalt mixtures requires deeper investigation considering fatigue, especially for mixes containing common road asphalt.
30. A linear regression model that refers to $DI_{\left(F\right)}$ ratio (Figure 13) could be used to successfully predict the degradation effect (to road salt) resulting in fatigue life decrease. The model determination coefficient was high (D, $R^{2}>0.89$) and very high (A, B, C—$R^{2}>0.97$).
31. According to applied linear regression models (Figure 13), degradation speed (fatigue) characterized by a model angle is generally the same among all mixes. It might be stated that all mixes, regardless of binder type, characterize the same trend for degradation speed considering various binder content levels.
32. Regarding regression models’ angles (Figure 13), it was observed that all models are parallel and shifted to each other. The angle is similar but $DI_{\left(F\right)}$ ratio not. Shift characterizes mix resistance due to road salt (fatigue) considering different binder types and content levels. Degradation vulnerability is technically the same for mix B, C and D (models are approximately superimposed). Mix A behaves differently–shifting via $DI_{\left(F\right)}$ ratio is high in comparison to other models. Such observation also proves point number 27 (WMA additives influence on mix vulnerability to road salt).
33. A linear regression model (Figure 14) referred to air void content level and $DI_{\left(F\right)}$ ratio are well correlated. The model determination coefficient was very high (A, B, C and D—$R^{2}>$0.93). It could be stated, that degradation (fatigue) caused by road salt also depends on air void content in the mix (except binder type and content).
34. Analyzing the correlation between air void content level and $DI_{\left(F\right)}$ ratio (Figure 14), it might be observed that mix B tended to be compacted more easily (resulting in lower air void content). Such mixes, characterized by slower air voids level increase via binder content level decreasing are more vulnerable to degradation processes via road salt impact—degradation speed acceleration (model’s angle) is higher.

## 5. Conclusions and Recommendations

The main aim of this study was to investigate the negative environmental impact (water and frost or road salt) on the asphalt mixture’s fatigue life based on two experimental methods related to appropriate impacts. In the article, four asphalt mixes (AC 22) were analyzed and compared. The investigation was performed successfully regarding proprietary methods usage, applied tests, developed ratio—$DI_{\left(F\right)}$ (fatigue degradation), and the results were discussed. The subject area was discussed as a complex problem (different mixes, binder content levels, production technology, and binder types). The manuscript proved that asphalt mixture degradation caused by environmental impacts (water-frost or road salt) considering fatigue is significant and could even be several dozen percent. Furthermore, it was proven that degradation via the following effects is a complex problem deserving of more profound attention. It is a noteworthy achievement regarding the actual state of knowledge.

It is also worth emphasizing that environmental factors affecting asphalt mixtures utilized in pavement construction (referring to fatigue—fatigue life, criteria, testing procedures, and material design guidelines) have not been taken into account so far. Attention should be paid to the subject area.

According to discussed results (the proposed laboratory testing procedure and information obtained), more essential conclusions and recommendations were drawn regarding the analyzed problem. They are listed below.

- Water and frost or road salt impacts caused the material degradation estimated by $DI_{\left(F\right)}$ ratio, referring to fatigue life decrease. The changes were significant—in the worst case, fatigue life reduction was equal to 28.7%. Environmental factors related to fatigue life should be considered, e.g., laboratory test procedures.
- Asphalt mixture degradation (fatigue) via water-frost or road salt depends on binder type (mainly), its content level (mainly), air void content level, and analyzed impact.
- Reducing binder content level in asphalt mixture recipes accelerates degradation (referring to fatigue life) caused by environmental effects.
- Air void content increase due to, e.g., compaction condition or low binder content level contributes to decreasing asphalt mixture resistance to environmental effects.
- Road salt impact caused a lower fatigue life reduction (up to 33%) than water and frost impact among all mixes.
- The mixtures containing highly modified 25-55/80 HIMA demonstrated the highest resistance to water-frost and road salt. It is stated that mixtures based on polymer-modified bitumen ensure higher durability (environmental resilience) and should be used in constructions subjected to difficult environmental and traffic conditions.
- Increasing polymer SBS content in asphalt matrix (difference between modified 25/55-60 and highly modified 25/50-80 HIMA asphalt usage) allows enhancing mix resistance (fatigue) to water-frost or road salt.
- The tested mixture (asphalt 35/50) made in hot technology distinguished up to 4.6 times higher resistance to the environmental factors (fatigue) than the alternative mixture (asphalt 35/50 WMA) made in warm technology. It is presumed that the presence of temperature-lowering additives (special waxes or chemicals) in the asphalt matrix increases the mixes’ susceptibility to environmental factors.
- Tested WMA mixes were very susceptible to environmental impacts due to fatigue. It is found that WMA mixture usage for pavements exposed to excessive exposure to de-icing agents (e.g., in mountains or roads with heavy traffic) is very limited. In such cases, it is recommended to use mixes containing common road asphalt (half better resistance).
- The developed degradation ratio $DI_{\left(F\right)}$ (fatigue degradation) usage enables practical and efficient analysis of mixture degradation caused by environmental factors. Ratio value enables to distinguish asphalt mixtures’ resistance and degradation speed changes (referring to fatigue) to mentioned effects considering different binder content levels.
- Environmental factors examination (considering fatigue) helps determine asphalt mixture susceptibility and allows to predict and choose the best solution for mix appliances in pavement construction due to fatigue life exhaustions and issues that could approach (cracking, chipping).

## Figures and Tables

**Table 1:** Mineral asphalt mixtures composition.

Mix Type	Mix Destination	Mineral Mix Composition	Binder Type	Binder Content
AC 22	binder course base course	16/22 gabbro grit—31.0% 11/16 gabbro grit—7.0% 8/11 gabbro grit—10.0% 4/8 gabbro grit—10.0% 2/5 gabbro grit—16.0% 0/2 gabbro crushed sand—18.0% 0/1 milled stone extender—8.0%	1. 35/50 WMA (WMA additive: N/A—restricted Lotos industry WMA formula) (common road asphalt)	3 identical content levels were analyzed for each binder type: 4.24% 4.03% 3.82%
			2. 35/50 (common road asphalt)	
			3. 25/55-60 (modified asphalt, polymer SBS)	
			4. 25/55-80 HIMA (highly modified asphalt, polymer SBS)	

**Table 2:** General fatigue test conditions.

Test Condition	Property	References
static diagram	4-PB-PR	PN-EN 13108-1:2016-07 [24] PN-EN 13108-20:2016-07 [25] PN-EN 12697-26:2018-08 [26] PN-EN 12697-24:2018-08 [27] WT-2 [22] Mackiewicz [28] Mączka [11]
load diagram	cyclically determined	
load cycle type	oscillatory cycle	
impulsive load shape	sinusoidal	
load condition	controlled displacement	
frequency	constant, 10.0 Hz	
temperature	constant, 10.0 °C	PN-EN 13108-1:2016-07 [24] PN-EN 13108-20:2016-07 [25] WT-2 [22] Sybilski [29] Judycki [30] Pszczoła [31] Leszczyńska [32] Haponiuk [33] Mączka [11,13]

**Table 3:** Comprehensive fatigue test results.

Mix	Binder Type	$V_{b}$ [%]	$V_{m}$ [%]	Variant	$E_{0,F\;}^{*}$ [MPa]	$E_{50\%,F\;}^{*}$ [MPa]	$N_{F,E50\%}$ [Cycles]	$DI_{\left(F\right)}$ [%]
A2	35/50 WMA	4.23%	4.10%	**ref**	9811	4906	311,340	
				**f-t**	8524	4262	239,465	**−23.09%**
				**rs**	8638	4319	256,645	**−17.57%**
A3	35/50 WMA	4.03%	5.08%	**ref**	10959	5480	217,004	
				**f-t**	9167	4583	165,402	**−23.78%**
				**rs**	9393	4697	176,493	**−18.67%**
A4	35/50 WMA	3.82%	6.22%	**ref**	10759	5380	149,994	
				**f-t**	8311	4156	107,053	**−28.63%**
				**rs**	8882	4441	117,192	**−21.87%**
B2	35/50	4.23%	4.20%	**ref**	9900	4950	411,640	
				**f-t**	8655	4327	322,025	**−21.77%**
				**rs**	9327	4663	395,943	**−3.81%**
B3	35/50	4.03%	4.44%	**ref**	10870	5435	296,816	
				**f-t**	9054	4527	224,923	**−24.22%**
				**rs**	10156	5078	279,894	**−5.70%**
B4	35/50	3.82%	5.00%	**ref**	10653	5326	161,401	
				**f-t**	7974	3987	115,563	**−28.40%**
				**rs**	9887	4944	145,906	**−9.60%**
C2	25/55-60	4.23%	4.00%	**ref**	9935	4968	1,456,839	
				**f-t**	9677	4838	1,398,566	**−4.00%**
				**rs**	9756	4878	1,436,444	**−1.40%**
C3	25/55-60	4.03%	5.08%	**ref**	10015	5007	928,584	
				**f-t**	9274	4637	810,726	**−12.69%**
				**rs**	9594	4797	879,084	**−5.33%**
C4	25/55-60	3.82%	5.59%	**ref**	9985	4993	497,310	
				**f-t**	8425	4213	399,351	**−19.70%**
				**rs**	9173	4587	455,498	**−8.41%**
D2	25/55-80 HIMA	4.23%	4.34%	**ref**	9310	4655	1,958,796	
				**f-t**	9229	4615	1,928,239	**−1.56%**
				**rs**	9194	4597	1,938,425	**−1.04%**
D3	25/55-80 HIMA	4.03%	4.97%	**ref**	10460	5230	1,032,285	
				**f-t**	10010	5005	972,412	**−5.80%**
				**rs**	10021	5010	1,009,574	**−2.20%**
D4	25/55-80 HIMA	3.82%	5.77%	**ref**	10208	5104	543,505	
				**f-t**	9289	4645	505,364	**−7.02%**
				**rs**	9511	4756	506,251	**−6.85%**

## Data Availability

Not applicable.
